# β-D-glucan Surveillance with Preemptive Anidulafungin for Invasive Candidiasis in Intensive Care Unit Patients: A Randomized Pilot Study

**DOI:** 10.1371/journal.pone.0042282

**Published:** 2012-08-06

**Authors:** Kimberly E. Hanson, Christopher D. Pfeiffer, Erika D. Lease, Alfred H. Balch, Aimee K. Zaas, John R. Perfect, Barbara D. Alexander

**Affiliations:** 1 Departments of Medicine and Pathology, University of Utah, Salt Lake City, Utah, United States of America; 2 Department of Medicine, Oregon Health Sciences University, Portland, Oregon, United States of America; 3 Department of Medicine, Duke University, Durham, North Carolina, United States of America; 4 Department of Pediatrics, University of Utah, Salt Lake City, Utah, United States of America; Research Institute for Children and the Louisiana State University Health Sciences Center, United States of America

## Abstract

**Background:**

Invasive candidiasis (IC) is a devastating disease. While prompt antifungal therapy improves outcomes, empiric treatment based on the presence of fever has little clinical impact. Β-D-Glucan (BDG) is a fungal cell wall component detectable in the serum of patients with early invasive fungal infection (IFI). We evaluated the utility of BDG surveillance as a guide for preemptive antifungal therapy in at-risk intensive care unit (ICU) patients.

**Methods:**

Patients admitted to the ICU for ≥3 days and expected to require at least 2 additional days of intensive care were enrolled. Subjects were randomized in 3∶1 fashion to receive twice weekly BDG surveillance with preemptive anidulafungin in response to a positive test or empiric antifungal treatment based on physician preference.

**Results:**

Sixty-four subjects were enrolled, with 1 proven and 5 probable cases of IC identified over a 2.5 year period. BDG levels were higher in subjects with proven/probable IC as compared to those without an IFI (117 pg/ml vs. 28 pg/ml; p<0.001). Optimal assay performance required 2 sequential BDG determinations of ≥80 pg/ml to define a positive test (sensitivity 100%, specificity 75%, positive predictive value 30%, negative predictive value 100%). In all, 21 preemptive and 5 empiric subjects received systemic antifungal therapy. Receipt of preemptive antifungal treatment had a significant effect on BDG concentrations (p< 0.001). Preemptive anidulafungin was safe and generally well tolerated with excellent outcome.

**Conclusions:**

BDG monitoring may be useful for identifying ICU patients at highest risk to develop an IFI as well as for monitoring treatment response. Preemptive strategies based on fungal biomarkers warrant further study.

**Trial Registration:**

Clinical Trials.gov NCT00672841

## Introduction

Invasive candidiasis (IC) is associated with high mortality, prolonged hospitalization and excess costs [1], [2], [3]. The majority of these infections are nosocomially acquired [4] and frequently complicate the management of intensive care unit (ICU) patients. Most invasive *Candida* infections are due to *C. albicans* followed in frequency by *C. glabrata*, a species that is often fluconazole resistant, and other non-*albicans Candida* species [4], [5], [6]. Antifungal prophylaxis reduces the overall incidence and associated mortality of IC [7], but a cost-effective approach requires targeting patients with the highest-risk of disease.

Establishing a definitive diagnosis of IC is difficult. The accompanying signs and symptoms are nonspecific and may not be present until the disease is advanced or disseminated. Standard microbiologic methods such as blood culture are time consuming and insensitive for early onset IC. Culture-based treatment is therefore delayed, which in turn fuels poor outcomes [1]. Unfortunately, empiric fluconazole based on the presence of fever has little clinical impact [8] and adequate empiric therapy is received by less than 30% of patients despite the availability of newer antifungal drugs with broad spectrum activity [9].

The limitations of empirical and culture-driven antifungal therapy have prompted investigation of alternative ICU strategies. These approaches include targeted prophylaxis [10], [11] and early treatment driven by the “*Candida* score” [12], [13]. The clinical benefit of risk-factor based regimes, however, remains largely unproven. 1, 3-β-D-glucan (BDG) is a fungal cell wall constituent found in many pathogenic fungi including *Candida*. The β-D-glucan test (Fungitell^™^, Associates of Cape Cod, Falmouth, MA) is approved by the United States Food and Drug Association (FDA) as an adjunct to the diagnosis of deep-seated mycoses. Serial measurement of fungal cell wall biomarkers with initiation of therapy in response to a positive test represents an exciting new approach to IC management. Additional proof-of-concept studies are required to establish BDG test performance and to assess the potential role of preemptive strategies in the ICU setting.

This is a single center, randomized, non-blinded, parallel group pilot study assessing the feasibility and utility of biweekly β-D-glucan testing as a guide for preemptive antifungal therapy in at-risk intensive care unit patients. We also assessed the outcome of biomarker driven preemptive therapy as compared to current practice (i.e., empiric treatment based on physician preference) for the management of IC. Overall, the purpose was to generate preliminary data to inform the design of a future, statistically powered multicenter clinical trial.

## Methods

The protocol for this trial and supporting CONSORT checklist are available as supporting information; see **Checklist S1** and **Protocol S1**.

### Ethics Statement

The study protocol was approved by the Duke University Institutional Review Board.

### Patients

Between June 2008 and January 2011, adult patients (≥18 years) admitted to the Surgical ICUs for at least 3 days, who were also expected to require ≥2 additional days of intensive care, were screened. Due to slow study enrollment, Medical ICU patients were also considered eligible beginning in August 2009. with intolerance to echinocandins, transaminases ≥10 times normal, visibly icteric serum, lung transplant recipients [14], and pregnant/lactating women were excluded. Additional exclusion criteria included: receipt of a systemic antifungal within the preceding week or a documented invasive fungal infection (IFI) at screening/baseline.

Eligible patients providing written informed consent were stratified according to APACHE II score (> or ≤20) and randomized in 3∶1 fashion to receive either active surveillance with preemptive anidulafungin or empiric antifungal therapy. Random allocation sequence was generated using free on-line software (Research Randomizer Version 3.0, http://www.randomizer.org). The sequence was generated by K.H. Randomization sequence was concealed from the study coordinators obtaining informed consent until the study arm was assigned by a research assistant.

### Active Surveillance/preemptive Therapy Group

BDG testing was performed at baseline and then twice weekly in the ICU. Results were reported within 24 hours, but baseline measurements were not used to inform initiation of preemptive therapy. Subjects with a subsequent BDG determination of ≥60 pg/ml were preemptively treated with a single course of intravenous anidulafungin (100 mg daily for at least 14 days). The dose and duration of anidulafungin was based on the FDA approved treatment schedule for candidemia. Blood cultures were obtained prior to initiation of preemptive therapy, with additional collections based on clinical indicators of infection. To improve sensitivity as a screening test, a positive BDG cut-off of 60 pg/ml was selected based on the observation that 81% of IC patients will have a positive test at this level [15]. BDG testing was otherwise performed per the manufacturer’s instructions.

To assess BDG kinetics on therapy, serum specimens were collected every other day while subjects were receiving anidulafungin. Kinetic sampling was batch tested retrospectively and these results were not available for treatment decisions by clinicians.

### Standard Care/empiric Therapy Group

Subjects in the empiric therapy group had BDG testing on the same schedule, but physicians remained blinded to the results. All antifungal treatment decisions were based on the providers’ clinical judgment, which is standard practice for non-transplant, non-leukemia/lymphoma patients at Duke University.

### Additional Study Procedures and Data Collection

An oral wash or endotracheal suction for *Candida* culture was obtained at enrollment. Subject demographics including risk-factors for IC were recorded at baseline. IC risk factors included: the presence of an indwelling central venous catheter, recent major surgery, receipt of broad spectrum antibiotics or total parenteral nutrition in the prior 3 days, ongoing hemodialysis, underlying diabetes, severe acute pancreatitis and/or an immunocompromised state [16]. Subjects were evaluated weekly until ICU discharge and/or for as long as they were receiving study drug. Clinical assessments included weekly physical examinations, medication review, and the evaluation of routine laboratory, microbiology, and radiographic results. The total length ICU stay, number of febrile ICU days, and overall disposition (alive or dead) at ICU discharge was recorded. Factors previously shown to be associated with false positive BDG tests were also evaluated (i.e., recent major surgery, hemodialysis, bacteremia, as well as receipt of commercial blood products and/or β-lactam antibiotics [17], [18], [19], [20], [21], [22], [23], [24], [25]). Adverse events (AEs) were regularly assessed and reported per the National Cancer Institute Common Terminology Criteria for Adverse Events version 3.0.

### Study Definitions

The criteria used to establish a diagnosis of proven or probable IC are displayed in **Table 1**. Other IFIs were classified according to the European Organization for Research and Treatment of Cancer/Mycosis Study Group (EORTC/MSG) criteria [26]. However, BDG results were not factored into the EORTC/MSG criteria. Cases of proven/probable IFI were adjudicated by a single reviewer (K.H.) blinded to group assignment and BDG results.

**Table 1:** Case Definitions for Proven and Probable Invasive Candidiasis (IC).

A. Proven IC
1. At least 1 blood culture growing *Candida*
2. *Candida* cultured from a normally sterile site (other than urine or sinus)
3. Histopathology showing invasive *Candida* forms in tissue
**B. Probable IC**
1. Fever (≥38.5°C) or hypothermia (<35.0°C) with leucocytosis (WBC ≥12 K) and/or hemodynamic instability (MAP <65) otherwise unexplained despite at least 3 days of broad spectrum antibiotics and both of the following:
a. *Candida* spp. isolated from at least 2 non-sterile sites (±3 days) and
b. No alternative microbiologic diagnosis.
2. Symptomatic *Candida* UTI defined as: ≥100, 000 cfu of *Candida* spp. in urine associated with pyuria (i.e., >5 WBC/hpf for non-neutropenic patients) and fever (≥38.5°C) or leucocytosis (WBC ≥12 K) not explained by another microbiologic diagnosis.
3. Endoscopically visualized esophagitis with biopsy exclusion of other causes and evidence of yeast on microscopy or *Candida* spp. grown in culture.

### Statistical Analyses

The sample size for this “pilot” study was not statistically based. Instead, a feasible sample size of 100 subjects was projected based on the number of eligible patients expected to be admitted to the ICU over a 1 year period. SAS (v9.1 SAS Institute Inc., Cary, NC) and R (v2.14.0 Foundation for Statistical Computing, Vienna, Austria) software packages were used for data analyses. Significance set at the 5% level.

The primary outcome measures of interest were the feasibility of preemptive antifungal therapy, BDG test performance and anidulafungin safety/tolerability in an ICU population. Feasibility was assessed with measures of protocol adherence. BDG test characteristics were evaluated with calculations of sensitivity (SN), specificity (SP), positive/negative predictive values (PPV and NPV) and positive/negative likelihood ratios (LR+ and LR-). Repeated measures logistic regression was applied to assess possible causes of false positive BDG results. Predicative variables included recent major surgery, renal replacement therapy, bacteremia, as well as receipt of commercial blood products and/or β-lactam antibiotics all within a week of BDG collection. Treatment emergent AEs were described with summary statistics.

Secondary outcomes included a comparison of the proportion of subjects developing a proven or probable IFI in the preemptive vs. empiric therapy groups as well as the effect of preemptive anidulafungin on serum BDG concentration over time. Clinical and microbiologic characteristics were compared using the Chi-squared or Fisher’s exact test for discrete variables and Mann-Whitney for continuous variables. The influence of antifungal treatment on BDG concentrations was assessed using non-parametric repeated measures ANOVA. A linear mixed effects model was also fitted, using a random subject intercept and a fixed treatment effect by study day. Bootstrap resampling was used to compare rates of glucan decline over time for preemptively treated versus untreated subjects.

## Results

**Figure 1:**
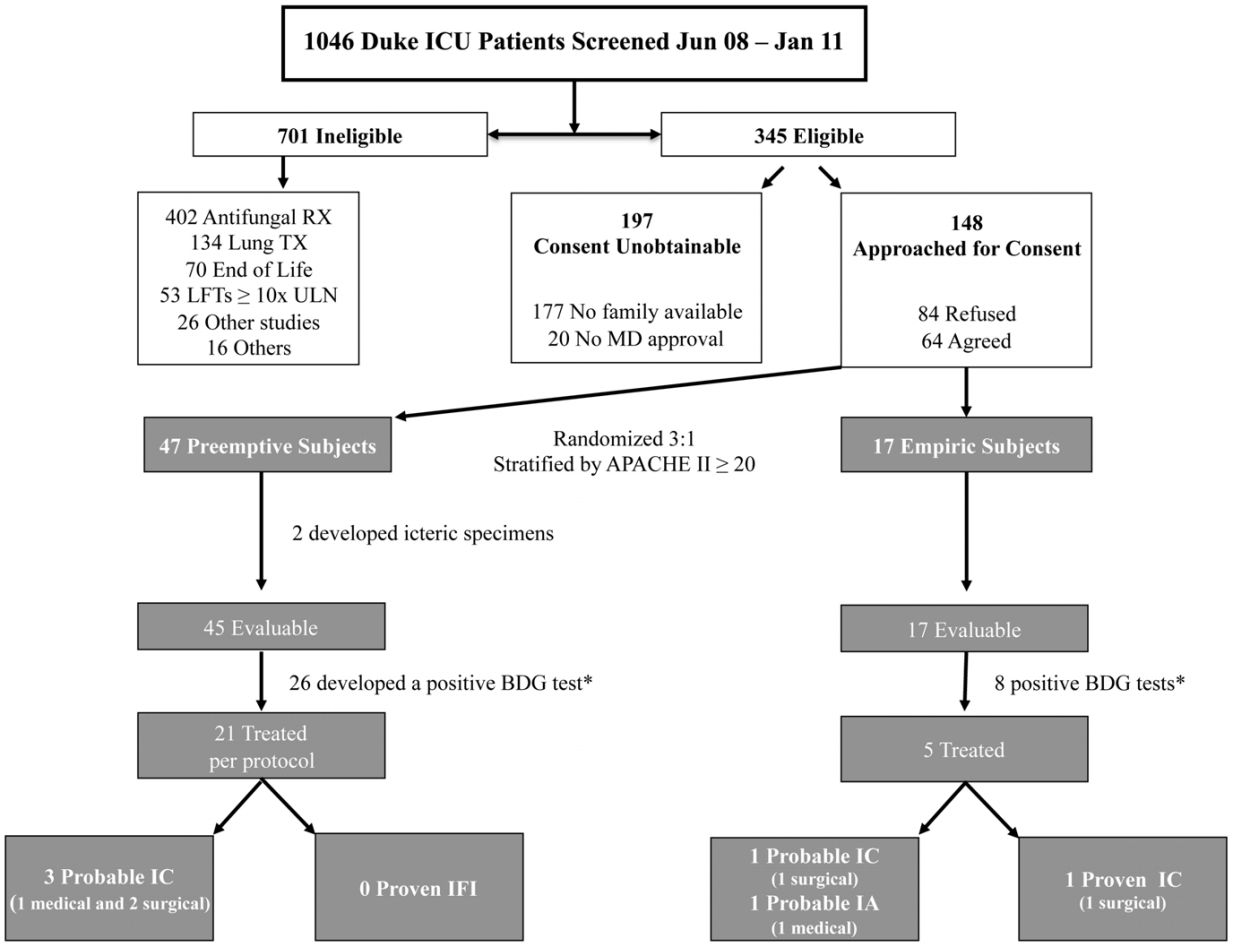
Study Subject Disposition. Abbreviations: RX = treatment; TX = transplant; LFTs = liver function tests; ULN = upper limit of the normal reference range; IC = invasive candidiasis; IFI = invasive fungal infection; IA = invasive aspergillosis; BDG = β-D-Glucan; medical vs. surgical = ICU location.*5 subjects in the preemptive group had a positive BDG test(s) but did not receive anidulafungin; 2 were BDG positive only at baseline/screening, 2 were transitioned to comfort care and 1 was treated empirically with antifungal therapy before development of a single positive test. All 5 subjects in the empiric therapy group that received systemic antifungal therapy had at least 1 positive BDG test around the time antifungal treatment.

More than 1000 ICU admissions were screened over a 2.5 year period. The trial was stopped in January 2011, short of the targeted 100 subject sample size, due to slow accrual. Subject disposition is illustrated in **Figure 1**. In all, 345 patients met study inclusion criteria, 64 of these agreed to participate and 47 were randomized to receive active BDG surveillance. Two subjects in the preemptive group developed grossly icteric sera which precluded reliable glucan measurements; therefore, these subjects were excluded from the BDG analyses. Approximately one-half of potentially eligible subjects (51%, 177/345) could not give effective informed consent due to the severity of their illness and did not have a designated medical decision maker available to discuss study participation.

### Study Subjects

Subject demographics are displayed in **Table 2**. The two study groups were generally well balanced with respect to baseline characteristics. The majority of subjects were surgical patients (83%, 53/64). However, there were no substantial differences in demographic characteristics or the total number of identifiable IC risk-factors between surgical and medical subjects (data not shown). The overall median APACHE II score was 14 (range, 5–25) and average length of ICU stay was 21 days (SD±12 days). Most subjects (92%, 59/64) had ≥3 IC risk factors documented at the time of study enrollment, 43 of 47 (92%) in the preemptive and 16 of 17 (94%) in the empiric group. Broad spectrum antibiotic usage (median 3 vs. 4 days duration), proportion of febrile ICU days (8% vs. 12%), length of ICU stay (median 19 vs. 15 days) and survival at ICU discharge (85% vs. 81% alive) did not differ between the preemptive and empiric groups, respectively. More subjects in the preemptive group received systemic antifungal therapy than did those in the empiric arm, but the difference did not reach statistical significance (53% [25/47] vs. 29% [5/17]; p = 0.16).

**Table 2:** Study Demographics*.

Parameter	Enrolled N = 64 [Total (%)]	Preemptive Group N = 47	Empiric Group N = 17
Median age (range in years)	60 (19–82)	58 (19–79)	60 (22–82)
Male sex	44 (68.8)	31 (70.0)	13 (76.5)
SICU location	53 (82.8)	37 (78.7)	16 (94.1)
Median APACHE II (range)	14 (5–25)	14 (6–25)	14 (5–25)
Central venous catheter	64 (100)	47 (100)	17 (100)
*Candida* colonization	51 (79.7)	38 (80.9)	16 (94.1)
Any Surgery	55 (85.9)	40 (85.1)	15 (88.2)
Intra-abdominal procedure	12 (18.8)	9 (19.1)	(17.6)
Broad spectrum antibiotic	45 (70.3)	33 (70.2)	12 (70.6)
Beta-lactam	42 (65.7)	30 (63.9)	12 (70.6)
Diabetes	29 (45.3)	23 (48.9)	6 (35.3)
Hemodialysis	5 (7.8)	4 (8.5)	1 (5.9)
Immunosuppressive therapy	9 (14.0)	8 (17.0)	1 (5.9)
Total parenteral nutrition	6 (9.4)	4 (8.5)	2 (11.8)
Blood Products	53 (82.8)	38 (80.8)	15 (88.2)
Acute Pancreatitis	1 (1.7)	1 (2.1)	0 (0)
ANC < 500	0 (0)	0 (0)	0 (0)
Median LOS (range in days)	17 (5–62)	19 (5–43)	15 (6–62)
≥3 Risk factors for IC	59 (92.2)	43 (91.5)	16 (94.1)
≥4 Risk factors for IC	40 (62.5)	31 (70.0)	9 (52.9)

* Subject characteristics and baseline invasive candidiasis (IC) risk factors were assessed beginning the week prior to study enrollment, percentages (%) are displayed unless otherwise specified. N = number, SICU = surgical intensive care unit; Colonization was defined as the isolation of *Candida* from any non-sterile site (e.g. oral wash, respiratory tract or urine) in the absence of clinical signs/symptoms of invasive disease; Broad spectrum antibiotic = systemic receipt of a drug with activity against both gram positive and gram negative organisms; Blood products = transfusion of packed red blood cells, platelets, cryoprecipitate, and/or fresh frozen plasma; ANC = absolute neutrophil count; LOS = length of ICU stay; IC = invasive candidiasis.

### Glucan Results

A total of 296 BDG tests were performed. Median time from ICU admission to the first BDG assessment was 8 days (range, 3–20). The median number of tests per subject was 4 (range, 1–13). In all, 34 subjects (55%) developed at least one positive BDG test (≥60 pg/ml) prior to receipt of systemic antifungal therapy and 26 (41%) had at least 2 sequential positive tests at this level. Nineteen (31%) subjects were BDG positive at screening/baseline and 2 of these were only positive at the time of study enrollment. Overall, quantitative BDG values ranged from <31 to 1994 pg/ml.

### Proven and Probable IC

There was no significant difference in the number of proven/probable cases of IC diagnosed between the preemptive and empiric treatment groups (6.4% [3/47] vs. 17.6% [3/17]; p = 0.47) (**Figure 1**). One proven case of IC (*Candida* peritonitis) was diagnosed in a surgical patient in the empiric therapy group. An additional 5 probable cases of IFI (4 IC and 1 invasive pulmonary aspergillosis) were identified during the study period, 3 in the preemptive and 2 in the empiric group**.** All 6 subjects with a proven or probable IFI had multiple sequential positive BDG tests and were treated with appropriate antifungal therapy. Median BDG values corresponding with proven/probable IFI were significantly higher than those obtained from subjects without a fungal infection (117 pg/ml vs. 28 pg/ml; p<0.001). Median BDG values (range in pg/ml) for subjects with proven, probable, or no IFI were: 103 (82–126), 112 (84–295), and 28 (<31–1994), respectively. The subject with *Candida* peritonitis had a single BDG test performed prior to the diagnosis. The result was positive (126 pg/ml) the day before symptoms prompted abdominal imaging followed by a diagnostic paracentesis. Culture of the peritoneal fluid grew *C. albicans* 3 days later.

### BDG Test Characteristics

**Figure 2:**
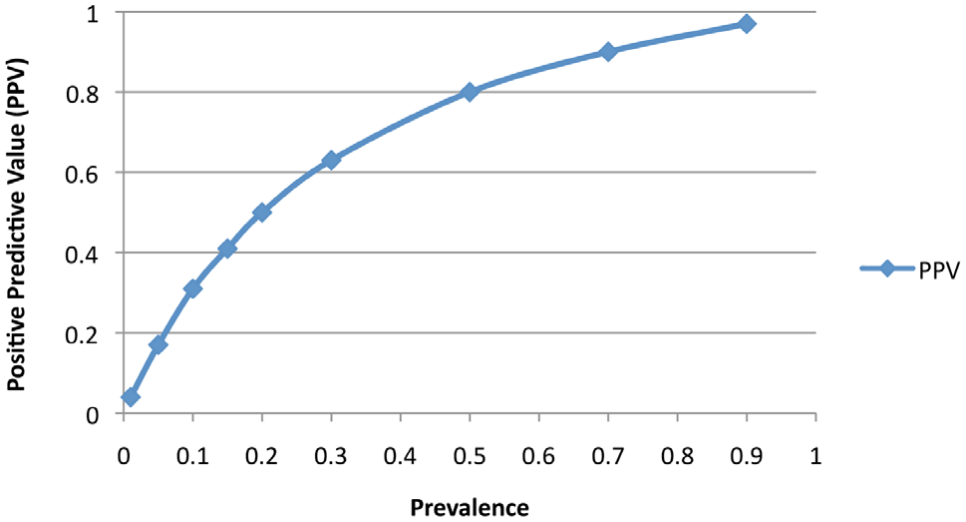
β-D-Glucan Positive Predictive Value as a function of varying Disease Prevalence. The positive predictive value of two sequential β-D-Glucan test results ≥80 pg/ml is plotted relative to increasing invasive candidiasis prevalence. Sensitivity and specificity have been fixed at 100% and 75%, respectively.

Composite definitions for proven or probable IFI were used as the “gold standard” for assessments of test performance**.** BDG had an overall sensitivity, specificity, positive, and negative predictive value of 100%, 50%, 17.6%, and 100%, respectively, when the a priori threshold of ≥60 pg/ml was used to define a positive test. **Table 3**. displays BDG test characteristics based on varying cut-offs. BDG performed best when 2 sequential specimens with values ≥80 pg/ml were required for a positive result (SN 100%, SP 75%, PPV 30%, and NPV 100%). This modified definition reduced the false positive rate (i.e. 1-specificity) from 50% using the ≥60 pg/ml threshold to 25%, without affecting clinical sensitivity. PPV was also calculated as a function of IC prevalence using the sensitivity and specificity estimates associated with the optimized test cut-off (**Figure 2**). Modest improvements in the PPV where observed when the disease prevalence was increased from 10% to 20%.

**Table 3:** 1,3-β-D Glucan (BDG) Test Characteristics.

BDG Test Result Cut-off (pg/ml)	SN %	SP %	PPV %	NPV %	LR(+)	LR(−)
Single ≥60	100	50	17.6	100	2	0
Single ≥80	100	58.9	20.7	100	2.4	0
Single ≥100	100	69.6	26.1	100	3.3	0
Sequential ≥60	100	67.9	25	100	3.1	0
Sequential ≥80	100	75	30	100	4	0
Sequential ≥100	50	78.6	20	93.6	2.3	0.6

SN = sensitivity; SP = specificity; PPV = positive predictive value; NPV = negative predictive value; LR (+) = positive likelihood ratio; LR (−) = negative likelihood ratio.

### False Positive BDG Tests

Overall, 45% (28/62), 37% (23/62) and 23% (14/62) of evaluable subjects had false positive BDG results when the ≥60, 80, and 80 pg/ml×2 thresholds were applied to case definitions of proven/probable IFI, respectively. There was no clear pattern as to the timing of these false positive BDG results (i.e., early [the first week] vs. late during the ICU admission).

Factors previously associated with false positive BDG results were common in the study population. The majority of subjects received B-lactam antibiotics (67.7%, 42/62) and/or commercial blood products (85.5%, 53/62) at some point during their ICU stay. In addition, 14 patients (22.6%, 14/62) developed a bacterial blood stream infection (BSI) while in the ICU (13 gram positive BSIs and 1 gram negative BSI). A single subject was treated with IVIG. This patient had the highest BDG values overall (range, 568–1994 pg/ml) and had received the infusion 2 days prior to the first positive test. Receipt of hemodialysis, however, was the only clinical factor statistically associated with elevated glucan levels (p<0.0001).

### Glucan Kinetics

**Figure 3:**
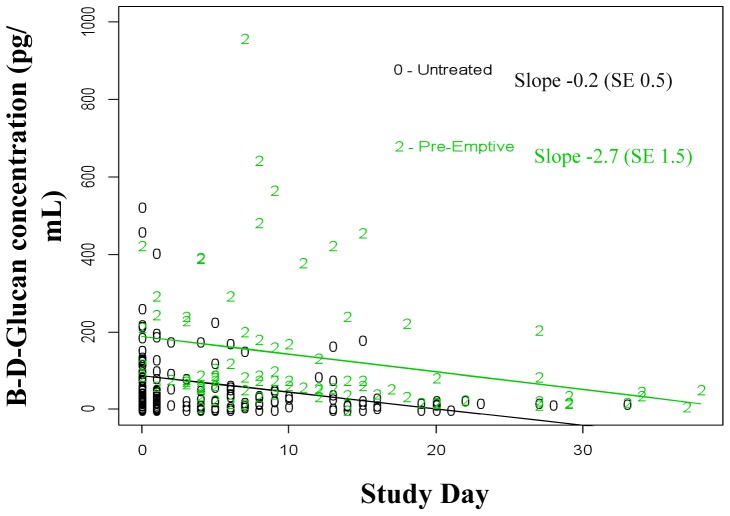
β-D-Glucan Concentrations Over Time in Subjects Receiving Preemptive versus No Antifungal Therapy. The antifungal treatment effect on glucan concentration over time was modeled as a linear trend. Abbreviations: 0 = subjects in the standard care group with at least one positive BDG test, but no systemic antifungal treatment; 2 = subjects in active surveillance group that were treated with preemptive anidulafungin; SE = standard error of the estimated glucan concentration slope.

Receipt of preemptive antifungal therapy had a significant effect on median glucan concentrations (p<0.001). However, BDG levels declined in both antifungal treated as well as untreated subjects over time (**Figure 3**). The slope of the decline was steeper for subjects receiving preemptive as compared to no antifungal therapy (slope −2.7 [SE 1.5] vs. −0.2 [SE 0.5]). The slope difference did not reach statistical significance (one sided p-value 0.06).

### Anidulafungin Safety and Tolerability

Twenty-one subjects received preemptive therapy for a median duration of 13 days (range, 2–27 days). In all, 10 (48%) subjects experienced 15 AEs that were possibly related to the study drug. Most AEs (80%, 12/15) were mild in severity, reversible, and did not require cessation of anidulafungin. The most common was elevated LFTs (9 of 21 subjects), followed by hypocalcemia and thrombocytopenia (2 of 21 subjects, each). No serious drug-related adverse events were observed during the study period.

### Feasibility Assessment

Preemptive protocol adherence was 86.7% (39/45). Four subjects received systemic antifungal therapy in spite of repeatedly negative BDG results (21.1%, 4/19), thus breaking the surveillance strategy. None of the 4 met study criteria for a proven or probable IFI. Antifungal therapy was prescribed for several reasons: 1 subject was diagnosed with polymicrobial sinusitis including yeasts, another developed a cutaneous yeast infection, and 2 were treated empirically for fever in the setting of other culture confirmed infections. Two subjects had preemptive therapy withheld despite non-baseline/screening BDG results of ≥60 pg/ml; but both had been transitioned to comfort care prior to receipt of the test results.

## Discussion

ICU patients have a higher incidence of invasive *Candida* infection than do those on general medical/surgical wards [5]. Antifungal prophylaxis has been proposed as a way to prevent IC in high-risk groups, but no consensus on the optimal strategy exists [27]. In terms of drug selection, the polyenes are limited by drug-associated toxicities and the azoles have been suspected of promoting a shift toward resistant non-*albicans Candida* species. Alternatively, the echinocandins have broad spectrum activity, are well tolerated with minimal drug interactions, and would likely be effective as prophylaxis in the ICU setting [28], [29].

Identifying ICU populations that are most likely to benefit from early interventions (i.e. the likelihood of IC exceeds 10% [30]) is difficult. Risk-factor based prediction rules and the “*Candida* score” consistently identify high-risk patients, but they remain imprecise. Risk classification schemes predicated on *Candida* colonization, for example, are potentially flawed by the inherent variability in culture acquisition. Previous ICU prophylaxis studies that based enrollment on multiple IC risk-factors closed prematurely due to difficulties identifying eligible subjects [31]. In addition, there is a general reluctance to enroll high-risk patients into placebo-controlled trials and/or to withhold empiric treatment from febrile patients already receiving broad spectrum antibiotics. Novel strategies are clearly needed.

Assays that detect fungal cell wall components in the blood of patients with early IFI are commercially available and have been used in Japan for many years. Attractive features of the Fungitell^TM^ BDG assay as a screening test are its sensitivity for the detection of early infection [32], [33], [34], [35] and high negative predictive value [36]. A few studies have evaluated the utility of serial BDG monitoring in the ICU [37], [38], [39], but none have used biomarker surveillance to inform early treatment decisions. The most recent report by Mohr et al., using the FDA approved cut-off of ≥80 pg/ml and institution-specific definitions of proven/probable IC, observed a BDG sensitivity and specificity of 91% and 57%, respectively [38]. When the number of positive specimens required to make a diagnosis of proven IC was increased to two or three, the specificity increased without affecting sensitivity. These observations are similar to ours, and when taken in the context of previous studies [32], serve to bolster the potential diagnostic value of sequential positive BDG results. A quantitative correlation between BDG levels and treatment response has also been reported [40], [41], which suggests that sequential monitoring on therapy could have prognostic value. We were unable to draw definitive conclusions about glucan kinetics following the initiation of antifungal therapy, likely due to our small sample size and variability in number of BDG measurements obtained per subject.

Potential causes of false-positive BDG results have been identified and many of these factors are common in the ICU [18], [19], [20], [21], [22], [23], [24], [35] . Receipt of hemodialysis was associated with elevated BDG levels in this study as well as in an earlier report involving lung transplant recipients at our institution [14]. These observations require further exploration given that renal elimination of BDG is not thought to be a major pathway of clearance and our dialysis membranes do not contain cellulose, a substance previously implicated in falsely elevated BDG levels [19]. We waited at least 3 days to begin active surveillance in an attempt to avoid other BDG cross-reactive substances present in early in admission [38] or following surgery [18], [20]. Using this delayed approach, significant proportions of subjects still had false-positive BDG results and were given anidulafungin. Changing the preemptive treatment threshold to require 2 sequential BDG values ≥80 pg/ml would reduce the number of patients treated by approximately half (a reduction from 59% [26/45] based on the current protocol to 24% [11/45]), without missing cases of proven/probable IC.

Overall adherence to the BDG surveillance protocol was good, with > 85% of preemptive subjects managed appropriately. Only 4 subjects assigned to the preemptive group received antifungal therapy despite negative BDG results and repeatedly negative results were used to safely withhold antifungal therapy in the majority of cases (78.9%, 15/19). Given the medical complexity of ICU patients, these observations suggest an acceptance of the BDG-driven strategy and a reliance on the negative predictive value of the test. BDG testing with present assays, however, is relatively expensive and labor intensive. Future studies incorporating biomarkers might consider alternative sampling schemes or the development of more facile assays that are amendable to the workflow of a routine clinical laboratory.

The main limitation of this study is the small number of subjects with proven/probable IC. Despite liberal inclusion criteria, we had a difficult time accruing patients not already receiving systemic antifungal therapy by day 3 of ICU admission. Targeted antifungal prophylaxis is routinely used for Duke solid organ transplant, peripheral blood stem transplant, high-risk leukemia/lymphoma and ventricular assist device patients. Reasons why potential subjects were already receiving systemic antifungal therapy was not documented as a part of the study screening process, however, we suspect that a significant proportion of ineligible ICU patients fell in to one of these categories. This may have biased study enrollment toward lower risk ICU subjects. In addition, we relied heavily on consent authorization obtained from patients’ legal representatives, but only half of all potentially eligible subjects had a designated surrogate available to discuss study participation during regular business hours. In the future, having the capacity to consent and screen both subjects in the evening and on weekends might modestly improve accrual. Perhaps more importantly, consideration should be given to initiating study enrollment/screening at the time of surgical or medical ICU admission, recognizing that some subjects will have a falsely elevated BDG level immediately following surgery. Antifungal therapy would then be withheld as a part of the protocol in selected subjects with a negative BDG test at baseline and during serial measurements.

In conclusion, this pilot study represents a first attempt at preemptive antifungal therapy based on the fungal biomarker BDG in busy tertiary care ICUs. Our results suggest that randomized studies of preemptive antifungal therapy in the ICU are feasible, but that these studies should ideally involve centers that do not routinely utilize fluconazole prophylaxis or employ protocols in which prophylaxis is held for selected patients with negative screening tests. Multicenter trials will ultimately be required to determine the efficacy, optimal algorithm, and cost-effectiveness of preemptive approaches for critically ill patients. Given the high mortality associated with delayed IC treatment and the ineffectiveness of empiric therapy based on fever, early preemptive approaches incorporating fungal biomarkers may have a substantial advantage.

## Supporting Information

Checklist S1 CONSORT Checklist.
(DOCX)

Protocol S1 Trial Protocol.
(DOCX)
