# Supplementary material for: β-D-glucan Surveillance with Preemptive Anidulafungin for Invasive Candidiasis in Intensive Care Unit Patients: A Randomized Pilot Study
Source: PLoS One. 2012 Aug 6;7(8):e42282. doi: 10.1371/journal.pone.0042282 (PMC3412848; doi:10.1371/journal.pone.0042282)
Supplement: Protocol S1 — Trial Protocol. (DOCX) [file pone.0042282.s002.docx]

**TITLE: Randomized Comparison of β-D-glucan Surveillance with Preemptive Anidulafungin versus Standard Care for the Management of Invasive Candidiasis in Surgical Intensive Care Unit Patients**

**REFERENCE NUMBER:** GA88517X

**PROTOCOL DATE:** September 28^th^, 2007

**PREPARED FOR:** Pfizer Inc

235 East 42^nd^ Street

New York, NY 10017-5755

**COMPOUND:** Anidulafungin (Eraxis™)

**REFERENCE NUMBER:** GA88517X

**PRINCIPAL INVESTIGATOR:** ____Kimberly Hanson, MD, MHS__

**TRIAL CENTER:** ___Duke University Medical Center_

**STUDY SYNOPSIS**

**Aims:**

**1)** Assess the clinical feasibility and utility of biweekly β-D-glucan testing as a guide for preemptive antifungal therapy in at-risk surgical intensive care unit (SICU) patients.

1. Evaluate the safety and tolerability of preemptive anidulafungin as compared to current practice (empiric antifungal therapy based on physician discretion) for the management of invasive candidiasis (IC).
2. Validate gene expression signatures predictive of IC.

**Hypotheses:**

**1)** Biweekly β-D-glucan surveillance will identify SICU patients with an invasive fungal infection (IFI) before clinical signs or symptoms develop.

1. Anidulafungin initiated in response to a positive β-D-glucan test (i.e. preemptive therapy) will be well tolerated, safe, and effectively treat patients early, before advanced infection is established.
2. Peripheral blood gene expression signatures obtained serially from at-risk patients can predict early IC.

**Study Design:** Single center, randomized, non-blinded, parallel group, comparative study.

**Sample Size:** 100 subjects; 75 in the active surveillance/preemptive therapy arm and 25 in the usual care arm.

**Study Population:** Adult patients admitted to Duke University Medical Center SICU at-risk for the development of IC.

**Study Drug:** Intravenous anidulafungin initiated in response to a positive β-D-glucan test for subjects in the active surveillance/preemtpive arm. Preemptive treatment will continue for at least 14 days or until ICU discharge (maximum 28 days), which ever is longer.

**Efficacy:** IFI assessments will be made weekly and at SICU discharge.

**Safety:** A data safety monitoring board will review adverse events every 4 months.

**Statistics:** β-D-glucan test characteristics will be described using sensitivity, specificity, positive and negative predictive values. The proportions of patients developing proven or probable IC in each group will be compared using Fisher’s exact test. The prevalence of treatment emergent adverse events will be described using summary statistics. For gene expression analysis, we will designate subjects diagnosed with a *Candida* bloodstream infection as case patients and those who remained uninfected and had negative B-D-glucan testing as controls. Decision tree analysis will be used to develop predictive signatures for candidemia versus no infection.

**Chart of Study Procedures**

| **Procedure** | **Screening/Baseline** | **Weekly** | **Bi-weekly** | **Treatment** | **SICU Discharge** |
| --- | --- | --- | --- | --- | --- |
| **Informed Consent** | **X** |  |  |  |  |
| **Clinical Information** | **X^a^** | **X^a^** |  |  |  |
| **Physical Examination** | **X** |  |  | **X^b^** |  |
| **APACHE II Score Assessment** | **X** |  |  | **X** |  |
| **IFI Assessment^c^** | **X** | **X** |  |  | **X** |
| **Medication Review** | **X** | **X** |  |  |  |
| **Β-D-Glucan^d^** | **X** |  | **X** | **X** |  |
| **Fungal Culture^e^** | **X** |  |  | **X** | **X** |
| **Fungal Susceptibility^e^** | **X** |  |  | **X** | **X** |
| **Β-hCG** | **X^f^** |  |  |  |  |
| **LFTs^g^** | **X** |  |  | **X** |  |
| **RNA collection^h^** | **X** |  | **X** | **X** |  |
| **Blood Culture^i^** |  |  |  | **X** |  |

^a^Demographics, baseline conditions, pertinent past medical history at baseline followed by weekly assessment for evidence of IFI and for IC risk factors.

^b^Obtain within 72 hours of 1^st^ dose of study drug and weekly there after while receiving anidulafungin.

^c^ Vital signs, clinical signs and symptoms, routine laboratory tests, culture results, radiographic findings, and/or biopsy reports indicative of IFI.

^d^ Only BDG results for subjects in the active surveillance/preemptive therapy arm will be reported to clinicians in real-time. BDG testing for subjects receiving usual care will be performed in batches, retrospectively. Subjects on anidulafungin will have the BDG test performed every other day until negative to determine kinetics on therapy. Samples collected for BDG kinetics will be batch tested retrospectively, therefore the results will not be reported to clinicians.

^e^ Oral swish and spit or ETS aspirate (from those subjects requiring mechanical ventilation) to assess for fungal colonization will be performed at baseline, after the completion of any systemic antifungal therapy, and at SICU discharge. Susceptibility testing to echinocandins and fluconazole will be performed on each isolate recovered. Results will not be reported to the care providers.

^f.^ Pregnant women will be excluded from the study.

^g^ LFTs will be collected at baseline and weekly on anidulafungin. LFTs include: total bilirubin, alkaline phosphatase, AST, and ALT

^h^ Hybridization to microarrays will occur in selected patients with positive blood culture results (cases) and in controls (no positive blood cultures, no empiric antifungal therapy).

^i^Two sets of blood cultures will be obtained within 24 hours of a positive BDG test result if not already collected as a part of clinical care.

**List of Abbreviations and Specialist Terms**

| **Abbreviation or**  **specialist term** | **Explanation** |
| --- | --- |
| AE | Adverse event |
| BDG | (1→3) β-D-glucan |
| BSI | Blood stream infection |
| CFR | Case fatality rate |
| CRF | Case report form |
| DSM | Data safety monitor |
| EORTC/MSG | European Organization for Research and Treatment of Cancer/Mycoses Study Group |
| ETS | Endotracheal suction |
| FDA | U.S. Food and Drug Administration |
| GU | Genitourinary |
| HSCT | Hematopoietic stem cell transplant; for this report, includes all blood- and marrow-derived stem cell transplants |
| IC | Invasive candidiasis |
| IFI | Invasive fungal infection |
| IRB | Institutional Review Board |
| IV | Intravenous |
| LFTs | Liver function tests |
| LLN | Lower limit of normal |
| OD | Optical density |
| RNA | Ribonucleic acid |
| RTT | Response-to-therapy |
| SAE | Serious adverse event |
| SICU | Surgical intensive care unit |
| TPN | Total parentral nutrition |
| SOT | Solid organ transplant |
| ULN | Upper limits of normal |
| WNL | Within normal limits |

**INTRODUCTION**

**Epidemiology of Invasive Candidiasis**

Fungal infections are devastating to human health and have lethal consequences for both immunocompromised and critically ill patients in the Intensive Care Unit. *Candida* species are now the 4^th^ most common cause of bloodstream infection in the United States (1), with the vast majority of these infections being nosocomially acquired(2). Most *Candida* blood stream infections (BSIs) are due to *C. albicans* followed in frequency by *C. glabrata*, a species that is generally less susceptible to traditional antifungal therapy (i.e., fluconazole). Despite the availability of newer antifungal drugs, the mortality attributable to invasive candidiasis (IC) remains extremely high (3). One reason that IC is so devastating is the difficulty in establishing a timely diagnosis. The clinical presentation of IC is highly variable and nonspecific, and standard blood culture is notoriously insensitive. In addition to the clinical impact, IC is a burden on the healthcare system, with estimates of increased costs on the order of $40,000 US dollars per episode(4;5).

Patients in the ICU have a higher incidence of *Candida* BSI as compared to those on general medical/surgical wards(1). Given that IC carries such a high associated morbidity, mortality, and cost, prophylactic antifungal therapy has been proposed as a way to prevent disease in high-risk patient populations. Azoles and polyenes have both been studied as prophylaxis in the ICU setting and were shown to decrease the incidence IFIs without impacting overall mortality(6-10). Individual prophylaxis studies, however, have varied widely with regard to patient selection and antifungal regimen. No consensus on the optimal preventative strategy currently exists, but experts have advocated some form of antifungal prophylaxis in situations where the rate of IC is expected to approach 10%(11).

**Issues Regarding Clinical Trials of Antifungal Prophylaxis**

Two key points of interest for future studies of IC prophylaxis are the choice of antifungal agent and the definition of patient risk. In terms of drug selection, the polyenes are limited by drug-associated toxicities and the azoles have been suspected of promoting a shift toward more resistant, non-*albicans* species(10). The echinocandins are the newest class of antifungal agents. The group is active against a broad range of *Candida* species, have relatively few drug-related toxicities/interactions, and would likely be effective as prophylaxis(12). As of yet, an echinocandin has not been studied for prophylaxis in the ICU setting. Many clinical risk factors for IC have been identified (**Table 1**), but their predictive value is uncertain(7). Risk-factor based prediction rules for the identification of individual patients at highest-risk for IC are typically complicated, too cumbersome and impractical to use in clinical practice, and may be too restrictive to use as enrollment criteria for clinical trials of antifungal prophylaxis. In fact, previous ICU prophylaxis studies that based inclusion on the presence of multiple clinical risk-factors closed prematurely due to difficulties identifying eligible patients in addition to the overall reluctance of clinicians to enroll high-risk patients in to a study with a placebo control arm.

**Preemptive Antifungal Therapy Based on Fungal Biomarkers**

β-D-glucan (BDG) is a cell wall constituent found in many pathogenic fungi including *Aspergillus* and *Candida* as well as *Fusarium*, *Trichosporon*, *Saccharomyces,* and *Acremonium*. Assays that are capable of detecting BDG in the serum of patients with IFI due to these organisms have been developed commercially. The β-D-glucan test (Fungitell^™^; Associates of Cape Cod, Falmouth, MA) is FDA approved as an adjunct to the diagnosis of deep-seated mycoses and fungemia. BDG analysis performed on serial serum samples obtained from 283 patients with acute myeloid leukemia or myelodysplastic syndrome who were receiving antifungal prophylaxis revealed that the test became positive a median of 10 days before the clinical diagnosis of IFI in 100% of patients(13). Absence of a positive test result had a 100% negative predictive value and a single positive test result had a specificity of 90%.

Primary prevention strategies that incorporate serial measurement of fungal biomarkers with initiation of antifungal therapy in response to a positive test represent an exciting new approach to IC management. Early initiation of appropriate antifungal therapy has been shown to improve clinical outcomes, but to date, the BDG assay has only been validated for patients with hematological malignancies and/or following transplantation. The performance of the test in the ICU setting has not been established. Poor specimen handling, hemodialysis using certain cellulose membranes, exposure to gauze products, some antibiotics, and recent receipt of albumin or immunoglobulin products may cause false positive test results in some patients(14-18). Since many of these potential sources of BDG cross-reactivity are present in the ICU population (**Table 2**), further investigation of the assay is warranted in this setting.

**Anidulafungin**

Until recently, the treatments available for serious fungal infections were limited to either amphotericin products (polyenes) or the azoles. Each class demonstrated significant limitations. The echinocandins are a new class of antifungal agents that appear to have several advantages over existing drugs. The echinocandins include 3 compounds: caspofungin, micafungin, and anidulafungin. Current evidence suggests that the three drugs are equivalent with regard to spectrum of activity, pharmacokinetics, drug interactions, and side effects. As a class, the echinocandins are active against *Candida* and *Aspergillus,* including species that are resistant to traditional antifungal agents. Primary or secondary drug resistance to the echinocandins is exceedingly rare. In addition, these compounds have demonstrated excellent clinical safety and efficacy as well as a more favorable toxicity profile than either amphotericin based products or the azoles(19).

Anidulafungin (Eraxis™, Pfizer) is the newest echinocandin to be approved by the FDA and has been studied for the treatment of esophageal candidiasis, candidemia, and deep-tissue IC. The drug works through the noncompetitive inhibition of (1,3)-β-D-glucan synthase(20), does not require dose adjustments in subjects with hepatic or renal impairment(21), and has demonstrated a low rate of adverse events in the phase 2/3 trials conducted to date. The drug is not a substrate, inducer, or inhibitor of cytochrome P450 and is therefore unlikely to affect the metabolism of other drugs that use these isoenzymes. Histamine-mediated symptoms have been reported during anidulafungin infusion including rash, urticaria, flushing, puritis, dyspnea, and hypotension, but are infrequent when the rate of infusion does not exceed 1.1 mg/minute. Laboratory abnormalities in liver function tests (LFTs) have also been seen in patients treated with anidulafungin, but the clinical significance of this observation is uncertain. Hepatic metabolism of anidulafungin has not been observed. Rather, the drug undergoes slow chemical degradation and is excreted primarily in feces(22).

Of the echinocandins, only micafungin has been studied as IFI prophylaxis(12). In a multi-center randomized trial, this agent was more effective than fluconazole (difference 6.5%, 95% CI: 0.9 – 12%) for the prevention of suspected, proven, or probable IFI during the Neutrogena period following hematopoietic stem cell transplant (HSCT)(12). The drug was safe and well tolerated as prophylaxis in this trial.

**Gene Expression Profiling to Diagnose Infectious Diseases**

The challenge for clinicians in diagnosing IC is both the delay in culture positivity as well as the relative insensitivity of blood cultures. The use of microarray technology to establish gene expression signatures unique to pathogens in parallel with established techniques may help quickly classify the cause of patient illness. Once the gene expression signatures are established, a rapid assay can be developed using multiplex PCR technologies. Such an assay would be used to target antimicrobial therapy and could be a valuable adjunct to pathogen based tests such as the BDG assay.

There is substantial evidence that different infectious agents trigger unique gene expression signatures in host immune effector cells both in vitro and in vivo. Gene expression in white blood cells provides direct analysis of early host response to invasive pathogens. Evaluation of these expression profiles provides not only a diagnostic opportunity, but also information regarding key elements of host response to a variety of pathogen classes. *In vitro* stimulation of human PBMCs with bacteria and bacterial products results in gene expression patterns that are pathogen specific(23). Members of our research group have identified gene expression signatures that can differentiate between typhus and paratyphus infections that cause a febrile illness that is otherwise clinically indistinguishable using a well-characterized Nepalese cohort (unpublished data). Researchers have also described gene expression patterns associated with viral(24) and fungal infections. These studies support the concept that identification of global changes in the gene expression patterns of PBMCs can be used to differentiate between major infectious causes of illness and can provide clinically meaningful classification of pathogens in patients.

**STUDY OBJECTIVES**

The main goal of this study is to evaluate the feasibility and safety of a preemptive antifungal treatment strategy, incorporating twice weekly BDG testing, as compared to an empiric approach (standard care) for the management of IC in at-risk adult SICU patients.

**Primary Objectives**

1. Determine the feasibility of preemptive antifungal treatment in an academic SICU setting
2. Define β-D-glucan test performance in the Duke SICU patient population
3. Evaluate the safety and tolerability of preemptive anidulafungin

**Secondary Objectives**

1. Compare the prevalence of IC between treatment groups (i.e., preemptive versus standard empiric therapy)
2. Compare the duration of SICU stay by treatment group
3. Compare the number febrile days by treatment group
4. Compare antifungal drug utilization between treatment groups
5. Compare the overall mortality rate between treatment arms at SICU and hospital discharge
6. Evaluate clinical response to preemptive or empiric antifungal therapy
7. Compare the prevalence of fungal colonization by group at baseline, at the end of antifungal therapy, and at SICU discharge
8. Determine the susceptibility of fungal pathogens identified (if any) and compare between groups
9. Assess the number of breakthrough IFIs in each management arm
10. Assess premature withdrawals from assigned strategy
11. Compare costs (antifungal drugs + diagnostic testing) between the 2 strategies
12. Obtain RNA from this well-characterized cohort of patients at-risk for the development of IC to derive gene expression signatures that characterize early candidemia

**STUDY DESIGN**

This is a single-center, randomized, parallel group, comparison of preemptive versus empiric antifungal therapy (e.g., standard care) for the management of suspected IC in SICU patients (**Figure 1**). Adult patients treated in the Duke SICUs for at least 3 days, and expected to require at least an additional 2 days of intensive care, will be approached for enrollment into the study. Patients providing informed consent will be stratified according to APACHE II score and randomly assigned in a 3:1 fashion to active surveillance with preemptive therapy or standard care. At Duke University, standard care is empiric antifungal therapy based on physician discretion.

**Active surveillance/ preemptive therapy group**

Subjects in this arm of the study will have serum drawn twice a week for BDG testing while in the SICU, with the results of the test reported to the managing clinicians in real-time. Subjects will receive intravenous (IV) anidulafungin should the β-D-glucan exceed 60 pg/ml on a single determination. A positive test cut-off of 60 pg/ml was selected based on the observation that 81% of patients with IC will have a positive test at this level(25). Preemptive therapy will continue for a minimum of 14 days or for as long as the subject remains in the ICU up to a maximum of 28 days duration.

If the BDG test turns positive and preemptive therapy is initiated, serum will then be collected every other day to assess the kinetics of the BDG test on anidulafungin treatment. Kinetic monitoring will continue until the test becomes negative at which time twice weekly assessments will be resumed and continue for as long as the patient remains in the SICU. Samples collected while patients are receiving anidulafungin will be batched and run retrospectively. Clinicians will not have these results made available for use in real-time. However, once preemptive anidulafungin therapy is completed and the drug has been stopped, biweekly monitoring with results reported to the medical team will be resumed. Subjects, however will not be restarted on anidulafungin should the test again turn positive after a course of preemptive therapy is completed.

**Standard care/empiric therapy group**

Patients in the standard care arm will also have biweekly blood draws for the BDG assay until ICU discharge, but the test will not be performed in real-time. Specimens collected from subjects in this arm of the study will be batched and tested retrospectively. The clinicians, therefore, will be blinded to the BDG results and can not use the assay to inform treatment decisions. Because BDG test characteristics in SICU patients are not known, an essential aspect of this study is our ability to assess test performance in the population of interest in an unbiased fashion. Patients in the standard care group may receive antifungal prophylaxis and/or treatment at any time based on the discretion of the treating physician.

**STUDY POPULATION**

**Inclusion Criteria**

Patients must meet all of the following criteria to be eligible for enrollment:

- Age ≥18 years
- Admission to the SICU or MICU for ≥ 72 hours and expected to stay and additional 48 hours
- IV access for administration of study drug
- Subject (or subject’s legal representative) able to give written informed consent

**Exclusion Criteria**

Individuals who have one or more of the following criteria will not be eligible for participation:

- History of hypersensitivity or intolerance to echinocandin antifungals
- Liver function test (ALT, AST, and/or total bilirubin)greater than 10 times the upper limits of normal (ULN)
- Pregnant or lactating women
- Treatment with systemic antifungal therapy within the preceding 7 days
- Documented IFI at baseline/screening
- Life expectancy less than 2 days or moribund

**CONSENT PROCESS**

Any one of the primary investigators may conduct the consent process. If the potential subject is awake, alert, and able to engage in discussion, the process will occur in the subject’s room. If the potential subject is sedated and/or unable to actively engage in the consent process for other reasons, discussion will be held with a legal representative, either in the subject’s room or in a private family conference room in the SICU. Approximately 30 minutes will be allocated for conducting the consent process, but may vary depending on the number or questions posed to the investigator. The subject or their surrogate will have up to 48 hours to consider whether or not to participate.

**Subject’s capacity to give legally effective consent**

A potential study subjects may not have the capacity to provide legally effective consent. Diminished capacity will not be assessed formally, but rather should be a reasonable clinical judgment made by the ICU care providers in conjunction with the study team. Factors to be considered include the subject’s mental status, sedative medications, and comfort level. These factors should be assessed by medication review and talking to the subject. Weekly reassessments of the subject’s capacity to give consent must be made. If a surrogate provided the initial consent, the subject should be reconsented as soon as their decisional capacity improves.

**STUDY MEDICATION**

**Rational for Dose Selection**

Subjects in the active surveillance arm who develop a single positive BDG test will receive preemptive anidulafungin intravenously. Preemptive therapy will include a loading dose of 200mg followed by 100mg maintenance therapy once a day. The loading and maintenance doses are derived from the FDA cleared schedule for IC and candidemia. No dosage adjustments are necessary for patients with hepatic or renal insufficiency, however, patients with LFTs ≥10 times ULN will be excluded from study participation. Isolated cases of hepatic dysfunction, hepatitis, or worsening hepatic failure have been reported in patients receiving anidulafungin, but a causal relationship to the drug itself has not been established.

**Duration of Preemptive therapy**

Preemptive therapy will continue until one of the following occurs:

1. A minimum of 14 days therapy (standard treatment duration for IC and/or candidemia*)*
2. Patient develops a proven or probable breakthrough IFI while receiving study drug (see appendix A for IFI criteria)
3. Patient develops unacceptable toxicity
4. The clinician or PI decides that it is not in the patients best interest to continue
5. Patient declines further study participation
6. Patient has received preemptive therapy for 28 days and has not yet been discharged from the SICU

**Drug Supplies**

Anidulafungin is manufactured by Pfizer, INC. Study drug will be provided by Pfizer, packaged in a single unit pack containing a vial of sterile, lyophilized, preservative-free powder (one 100mg vial) and a 30 ml diluents vial. The study drug supply will be maintained in the Duke University Medical Center Investigational Drug Pharmacy until administration. Storage will be as per the package insert. The medication will be reconstituted, mixed, and dispensed by the study pharmacist as directed on the product label. The pharmacist will be responsible for maintaining a dispensing log and receiving used bottles returned by nursing personnel after study drug administration.

**Drug Labeling**

Each bottle of study medication will be labeled with the study protocol number, drug name, strength, and batch number. Spaces will be provided for recording the patient’s initials, study ID number, date of dispensing, and amount to be administered.

**Drug Administration**

Study drug will be administered through IV infusion by nursing personnel according to the package insert. The rate of infusion will not exceed 1.1 mg/minute. If a study participant is discharged from the hospital prior to completion of preemptive therapy (minimum 14 days of treatment), they may have the study drug discontinued or continue daily administration of study drug by a visiting nurse at the discretion of their care provider. The sponsor will provide study drug to complete a total of 14 days, but will not cover the cost of home health care/infusion.

**Drug Accountability**

The Investigators will maintain accurate records of dates, quantities, lots of product(s) received, to whom dispensed (patient-by-patient accounting), and accounts of any product accidentally or deliberately destroyed. The Investigators will retain all unused or expired product. At the conclusion of the study, all unused drug supplies will be returned to the sponsor. An overall summary of all drug supplies received, used, wasted, and returned will be prepared at the conclusion of the study.

**Concomitant Medications**

There are no drugs that are contraindicated with anidulafungin. However, for study purposes, other systemic antifungal agents including amphotericin products, azoles, and/or investigational antifungal drugs may not be given during study drug dosing. If a patient in the preemptive therapy arm requires one of these agents, the patient must be discontinued from the study.

**SCHEDULE AND DESCRIPTION OF STUDY PROCEDURES**

**Identification of Potential Study Subjects**

A waiver of HIPPA and consent will be requested from the Duke University Medical Center IRB to review the SICU patient census daily and identify adults who have been in the unit for at least 3 days and who are expected to stay for an additional 2 days.

**Stratification and Randomization**

Patients who meet inclusion/exclusion criteria and provide written informed consent will be stratified into 2 groups based on APACHE II score. Group 1 will have APACHE II scores ≤ 20 and group 2 > 20. Subjects within each APACHE II group will then be assigned to either active surveillance with preemptive anidulafungin based on a positive BDG test or standard care using a 3:1 randomization schedule (approximately 75 subjects in the preemptive group and 25 in the standard care group). The study coordinator will assign each patient to the next available randomization number which will be generated using randomization software. Patient study identification (ID) numbers will also be assigned sequentially starting with Patient No. 1. Dropouts will not be replaced.

**Screening/Baseline**

Procedures must be performed within 3 days prior to randomization:

- **Signed informed consent prior to the performance of any study-specific procedures.**
- Complete medical and medication history, including a prior antifungal therapy review.
- Complete physical examination including vital signs.
- Liver function tests will be drawn if not already done as a part of standard care in the preceding 3 days.
- Calculate APACHE II score (<http://www.sfar.org/scores2/apache22.html>)
- Oral wash or ETS for semi-quantitative fungal culture with susceptibility testing.
- B-HCG test for women of childbearing age if not already done as a part of standard care (<60 years old)

**At Randomization**

- Stratify by APACHE II score
- Concomitant medications will be recorded.
- A serum red-top tube for BDG testing (3.5 ml) and a PAX Gene tube (5 ml) for RNA analysis will be obtained. *The PAX Gene tube must always be collected second due to processing requirements.

**All Subjects**

The following procedures will be performed for all study subjects for the duration of the SICU stay:

- Serum red-top BDG and a PAX Gene tubes will be obtained 2 times/week (generally Monday and Thursdays) with the routine early morning blood work by the SICU nursing staff. Both phlebotomy tubes will be sent directly to the Duke Clinical Microbiology Laboratory. BDG results will be made available in real-time (within 24–72 hours) via a phone call directly to the clinician (surgical house staff and unit pharmacist) caring for the study subject assigned to preemptive therapy. BDG specimens obtained from patients assigned to empiric therapy (standard care) will be batched and tested at the end of study and the clinicians will not be made aware of the results. All RNA analyses will also be performed retrospectively for research purposes only.

*Patients in the active surveillance/preemptive therapy arm may have BDG testing performed on other days if the providers feel that the result would immediately impact initiation of antifungal therapy. The test will be available daily Monday through Friday.

- IFI assessments will be made weekly (EORTC/MSG criteria for proven or probable IFI will be applied [**Tables 3 & 4**]) and documented on the CRF.
- Supporting evidence of IFI obtained as part of routine care will be recorded weekly (i.e. routine laboratory testing, microbiology and blood culture results, imaging studies, biopsy reports) and documented on the CRF.
- Clinical risk factors for IC will be documented weekly (**Table 1**) and documented on the CRF.
- Factors known to be associated with false positive BDG tests will be documented weekly (**Table 2**) and documented on the CRF.
- Concomitant antimicrobials and immunosuppressive drugs will be documented weekly on the CRF.
- AEs will be recorded weekly and documented on a dedicated CRF.

**INITIATION OF PRE-EMPTIVE OR EMPIRIC THERAPY**

- APACHE II score recalculated based on laboratory and clinical features in preceding 24 hours (http://www.sfar.org/scores2/apache22.html).
- Complete physical examination including vital signs will be obtained within 72 hours of study drug initiation.
- Assess and document whether patient meets EORTC/MSG criteria for proven or probable IFI.
- Laboratory evaluation (LFTs will be obtained if not already done in preceding 3 days).
- Oral wash or ETS for semi-quantitative fungal culture with susceptibility testing will be performed.
- Supporting evidence of IFI obtained as part of routine care will be documented.
- Positive BDG test will prompt two sets of blood cultures to be obtained within 12 hours, if not already collected a part of routine care. Culture results will be made available for clinical care.
- Concomitant medications will be documented.
- AEs will be recorded.

**WHILE ON PREEMPTIVE THERAPY**

- Serum for BDG will be collected every other day until negative. Once the test becomes negative, twice weekly surveillance will be resumed.
- Complete physical examination including vital signs once weekly.
- Assessment for breakthrough IFI as defined by EORTC/MSG criteria.
- LFTs once weekly.
- AEs will be recorded and drug tolerability assessed at the time of the physical examination.
- PAX Gene tubes will continue to be obtained 2 times/week.
- Concomitant medications will be documented weekly.

**END OF PREEMTPIVE THERAPY**

- Clinical response to therapy will be recorded on the CRF (**Table 5)**.
- Subjects will again be assessed for EORTC/MSG criteria for proven or probable IFI.
- Oral wash or ETS for semi-quantitative fungal culture with susceptibility testing.

**EMPIRIC THERAPY**

- Antifungal agent will be documented.
- APACHE II score recalculated based on laboratory and clinical features in preceding 24 hours.
- EORTC/MSG criteria for proven or probable IFI will be determined and documented on the CRF.
- Supporting evidence of IFI obtained as part of routine care will be documented.
- AEs will be recorded weekly.
- Oral wash or ETS for semi-quantitative fungal culture with susceptibility testing will be collected at the beginning and end of empiric therapy.

**FOLLOW-UP (SICU DISCHARGE)**

All study subjects will be followed until SICU discharge or for as long as they are receiving study drug, whichever is longer.

- Assess whether patient has evidence IFI or meets EORTC/MSG criteria for a new proven or probable IFI at SICU discharge.
- Assess mortality at SICU and hospital discharge. Cause of death and date of death will be documented.
- Oral wash or ETS for semi-quantitative fungal culture with susceptibility testing will again be obtained at SICU discharge.

**DIAGNOSTIC TESTS**

Serum for BDG and a PAX Gene tube will be obtained along with the routine morning SICU blood work twice weekly on Monday and Thursday. Samples will be sent to the Duke Clinical Microbiology Laboratory. Serum for BDG will be processed by the study technologist within 6 hours of collection. BDG will be performed by the study technologist and the results reported immediately to the SICU for patients in the active surveillance arm. Serum from patients in the standard care arm will be stored at -70°C and tested at the completion of the study. The BDG assay will be performed as per manufacturer’s instructions, using a positive cutoff of ≥ 60 pg/mL. Other study related laboratory tests (i.e., LFTs) will be performed by the Duke Clinical Laboratories. Blood cultures, surveillance fungal cultures, and susceptibility testing will be performed by the Duke Clinical Microbiology Laboratory as per standard operating procedure. All but the surveillance fungal culture will be reported in the medical record for clinical use. The PAX Gene collection system can remain at room temperature for up to 72hrs, samples will be processed by Dr. Aimee Zaas and all RNA analyses will be performed retrospectively after study completion.

**RNA ANALYSIS METHODS:**

For initial mining of gene expression data, subjects with known positive blood cultures (cases) and those with known negative blood cultures and negative BDG testing (controls) will be selected for initial gene expression studies. RNA will be extracted from PAXgene tubes using standard methodology (RNeasy, Qiagen) and subject to globin reduction (Ambion) by standard methodology. The Duke University Microarray Core will perform quality assurance and hybridization to Operon Human 4.0 Microarrays per standard methodology ([www.microarray.duke.edu](http://www.microarray.duke.edu)). We will then use the decision tree approach to find gene expression patterns that are correlated with candidemia and absence of candidemia to determine the predictive capability of those gene patterns as a diagnostic test.

**DESCRIPTION OF STUDY END POINTS**

**Primary Endpoints**

1. Feasibility of preemptive monitoring strategy.
2. β-D-glucan test performance in the stand care (empiric therapy) group.
3. Safety and tolerability of preemptive anidulafungin.

**Secondary Endpoints**

1. Prevalence of IC by treatment group (i.e., preemptive therapy versus standard care).
2. Duration of SICU stay by treatment group.
3. Number febrile days by treatment group.
4. Antifungal drug utilization by treatment group.
5. Overall mortality rate between treatment arms at SICU and hospital discharge.
6. Response to preemptive or empiric antifungal therapy will be assessed.
7. Fungal colonization rate by group at baseline, at the end of antifungal therapy, and at SICU discharge.
8. Susceptibility of fungal pathogens identified (if any).
9. Number of breakthrough IFIs by group.
10. Number of premature withdrawals from assigned strategy.
11. Costs (antifungal drugs + diagnostic testing) by group.
12. Gene expression signatures related to early candidemia (prior to blood culture positivity) and late candidemia (after blood culture positivity) and no candidemia (negative blood culture and negative B-D-glucan testing).

**END POINT DEFINITIONS**

**Feasibility**

The feasibility of the preemptive monitoring and treatment strategy will be assessed by evaluating adherence to the surveillance protocol in combination with the number of instances where BDG was ordered off schedule to inform treatment decisions and/or systemic antifungal therapy was initiated despite a negative BDG result.

**Invasive Fungal Infection**

The European Organization for Research and Treatment of Cancer/Invasive Fungal Infections Cooperative Group and the National Institute of Allergy and Infectious Diseases Mycoses Study Group (EORTC/MSG) established standard definitions for IFIs for use in clinical research(26). These criteria were designed for immunocompromised patients with cancer and hematopoietic stem cell transplant (HSCT) recipients. Although not developed for ICU patients specifically, these criteria will be applied as a way to standardize the assessments and classification of IFI in study subjects (**Tables 3 and 4**)

**Invasive Candidiasis**

IC will be defined as proven deep tissue *Candida* infection or candidemia.

**Breakthrough Invasive Fungal Infection**

Breakthrough IFIs will be defined as a proven or probable fungal infection that is not present when preemptive or empiric is initiated, but develops and is diagnosed later, after at least 72 hours of antifungal therapy.

**Clinical Response**

Response to empiric therapy will be defined as the resolution of fever and/or other attributable symptoms or signs of IFI after the initiation of systemic antifungal treatment. Response to preemptive therapy will be defined as normalization of the BDG test and resolution of fever and/or other attributable symptoms or signs of IFI (if these were present at the time the BDG test became positive) after the initiation of anidulafungin treatment.

**ASSESSMENT OF SAFETY**

Safety will be assessed by adverse events (AEs), physical examination, measurement of vital signs, and through the use of clinical laboratory tests obtained either as a part of routine care or once weekly during active preemptive treatment.

**Adverse Events**

AEs are defined as any untoward medical occurrence that develops during a clinical trial. An AE can be any unfavorable and unintended sign, symptom, or disease temporally associated with the use of a medicinal product, regardless of the relationship to the study drug. Each AE will be recorded on a separate adverse event case report form (CRF). Concomitant medications will be documented by generic name. Laboratory abnormalities will be described. Medications taken to relieve symptoms of the AE will also be recorded in addition to the outcome. AEs will be reported per the National Cancer Institute Common Terminology Criteria for Adverse Events (NCI CTCAE) version 3.0. The Principal Investigator will determine the severity of events and grade the event as follows:

**Grade 1:** Mild AE

**Grade 2:** Moderate AE

**Grade 3:** Severe AE

**Grade 4:** Life-threatening or disabling AE

**Grade 5:** Death related to AE

**Serious Adverse Events**

All serious AEs (SAEs) will be followed until resolution and/or until a cause is identified. SAEs include the following:

- Death
- A life-threatening adverse drug experience
- Inpatient hospitalization or prolongation of an existing hospitalization
- A persistent or significant disability/incapacity (defined as a substantial disruption of a person’s ability to conduct normal life functions)
- A congenital anomaly/birth defect
- An important medical event that (while it may not result in death, be life-threatening or require hospitalization) may jeopardize the patient and require medical or surgical intervention to prevent one of the outcomes listed in this definition

**Reporting Serious Adverse Events**

Any SAE occurring during the course of the study, considered drug related, will be reported by fax within 24 hours to the sponsor. In addition, these SAEs will be reported to the Duke Institutional Review Board (IRB) within a timely manner according to the IRB requirements. Study drug related SAEs will also be monitored by the Data Safety Monitor (DSM) throughout the study and reported to MedWatch. The DSM may at any point temporarily stop the study while he/she evaluates the impact of the SAE(s).

**DISCONTINUATION FROM STUDY**

The following occurrences shall result in the discontinuation of a patient from the clinical study:

- Patients who develop a Grade 4 or Grade 5 SAEs considered possibly, probably, or definitely to be related to study drug.
- Patients who decide not to participate further in the clinical study.
- If, in the investigator’s or clinician’s opinion, it is in the best interest of the patient to discontinue participation in the study.
- Patients in the active surveillance/preemptive therapy arm who are started on systemic antifungal therapy in the absence of a positive BDG test or without evidence a new proven or probable IFI by EORTC/MSG criteria.

All patients who withdraw or discontinue due to reasons other than withdrawal of consent will be followed for the endpoints of mortality at SICU and hospital discharge.

**DATA SAFETY MONITOR**

A DSM consisting of an Infectious Diseases physician will review the study every 4 months. The DSM can recommend early terminations to assure the safety of study participants or for efficacy in order to limit the number of patients monitored by a strategy that is less effective. However, specific statistical stopping rules will not be applied. The DSM will make recommendations regarding adaptations to the study if needed after the review of interim analysis results.

**STATISTICAL METHODOLOGY**

**Sample Size**

Targeted enrollment is 100 adult SICU patients. This study will not be powered to show a difference in the efficacy of preemptive management versus standard care due to single center population constraints in addition to cost limitations. Sample size calculations were therefore based on the number of adult patients expected to be admitted to the SICU over a 1 year period who are likely to meet inclusion criteria (n=700). Based on prior experience, it is reasonable to expect an accrual of 100 patients in a year. Using a 3:1 randomization strategy, 75 will be assigned to active surveillance and 25 to standard care. We estimate that 25% of patients will develop a positive test(13;25;27) and 19 will receive anidulafungin. These numbers should provide sufficient data for a preliminary feasibility evaluation of the preemptive strategy.

**Statistical Analyses**

Statistical analysis will be performed using SAS software. P values and 95% confidence intervals will be 2-tailed and statistical significance set at the 5% level unless otherwise specified.

*Demographics and Baseline Characteristics*

Demographic and baseline characteristic information will be summarized and compared using the Chi-squared test for discrete variables and one way ANOVA for continuous various to assess any imbalance between treatment groups.

*β-D-glucan Test Characteristics*

β-D-glucan test characteristics, using data collected from standard care group, will be described using sensitivity, specificity, positive and negative predictive values. Proven and probable cases of IFI, as defined by EORTC/MSG criteria (**tables 3 and 4**), will constitute the standard for comparison.

*Feasibility Analysis*

Feasibility will be a description of preemptive protocol adherence, BDG test requests off schedule, and the number of patients withdrawn from the preemptive arm due to the initiation of empiric systemic antifungal therapy in the absence of a positive BDG test.

*Efficacy End-Points*

Development of IFI during the study period will be assessed by the principal investigator (PI) and a second blinded investigator using predefined EORTC/MSG criteria for proven or probable disease. The study period is defined as the time the patient was cared for in the SICU. The proportions of patients developing IFI, and IC specifically, in each group will be compared using Fisher’s exact test. The median ICU stay and number of febrile days while in the ICU will be compared using the Kruskal-Wallis test. Patient mortality, overall and attributed to IFI, will also be summarized by group. Response-to-therapy (RTT) will be determined by the PI and a blinded second investigator. The RTT population will consist of all patients who were underwent BDG monitoring for at least 3 days and received at least 1 dose of systemic antifungal therapy.

*RNA Analyses*

Initial signature development cohort: RNA from patients with blood cultures growing *Candida* and an equal number of patients with negative blood cultures and negative BDG testing (controls) will be chosen for signature development. A decision tree approach used previously by our group will be used to find gene expression patterns that are correlated with candidemia to determine the predictive capability of those gene patterns as a diagnostic test(28;29). Once we identify a large number (100-300) of gene expression patterns that significantly correlate with the illness classification in our initial cohort, we will then identify the best combination of 3-5 patterns for discriminating between those with candidemia and those with no infection. Once the best expression patterns are identified, we will perform cross validation analysis in which we construct a decision tree with the best gene patterns using all of the samples except for one “held out” sample. We can then use the decision tree model to predict the classification of the “held out” sample as an honest test of the accuracy of our predictive model and to guard against over-fitting. We plan to define host gene expression patterns in response to candidemia using an initial cohort of up to 30 persons with candidemia and up to 30 persons with no infection. This sample size has 90% power to detect at least a 2-fold change in expression levels in the 70% least variable genes with an alpha of 0.001(30). After establishing robust gene expression signatures that classify individuals as having a “candidemia” or “no infection” we will test the generalizability of these profiles on the remaining patients presenting to the intensive care unit at risk for invasive candidiasis. We will not know beforehand the etiology of their symptoms.

**Safety**

All patients who receive at least 1 dose of study medication will be included in the safety analysis. The prevalence of treatment emergent adverse events will be described using summary statistics and compared used the Chi-square test.

**Missing Data**

No missing data imputation will be performed in the primary analysis. If a variable is missing in 50% of the patients to be included in the primary analysis or secondary analyses, then dropping the variable from analysis may be considered. If dropouts are numerous or unbalanced among treatment groups, safety assessments among dropouts will be listed and summarized by treatment group and reason for discontinuations (grouped as due to AE, withdrew consent, lost to follow-up, death, etc.).

**Censoring**

Subjects who withdraw consent are the only subjects who will be censored; date of withdrawal will be used as time point for censoring. All other patients who withdraw or discontinue for other reasons, including AEs, will be followed for mortality at the time of SICU discharge as well as at hospital discharge.

**Data Analysis**

Data Analysis will be performed after completion of data entry, database closure, and verification of database accuracy. All primary and secondary analyses will be performed on the intent-to-treat basis.

**INTERIM ANALYSES AND STOPPING RULES**

The planned interim analysis of key safety and endpoint data will be performed by the DMC every 4 months. It is unlikely that there will be a sufficient numbers of patients to claim statistically significant differences for the secondary IFI or mortality endpoints. The primary objective of these analyses will be to view the data for safety outcomes and to look for differences in mortality and the development of IFI between the 2 arms. Other objectives are to review patient recruitment, compliance with study protocol submission of data forms, and other factors that reflect study progress. If significantly large and important differences are observed in mortality or IFI prevalence between the 2 arms, the DMC may recommend the randomization of patients be stopped, or if the difference observed is indicative of efficacy, the design and conduct of the trial may be modified to recruit more patients and to design the trial to evaluate differences in mortality between the 2 arms.

**DIRECT ACCESS TO SOURCE DATA/DOCUMENTS**

The patients (or their legal representative) must also allow access to the patients’ medical records. They will be informed of this and will be acknowledging their agreement when signing the informed consent. Access to patient records and source data must be made available to the IRB, auditors, regulatory authorities, and the sponsor as appropriate.

**QUALITY CONTROL AND QUALITY ASSURANCE**

All data collected on the study CRFs will be entered into a password protected electronic ACCESS database. Every 4 months, verification of data within the electronic data base will be compared to source documents and CRFs to insure the accuracy, consistency, completeness, and reliability of the data.

**ETHICS**

The study will be conducted in compliance with the protocol, good clinical practice, and all applicable regulatory requirements. The study must be approved by the IRB prior to screening in accordance with Duke policies and guidelines.

**INFORMED CONSENT**

Properly executed written informed consent, in compliance with 21 Code of Federal Regulations 50 and International Conference on Harmonization (ICH) guidelines, shall be obtained from each patient or patient’s authorized legal representative prior to entering the patient into the trial. The Investigator shall provide a copy of the signed informed consent to the patient or patient’s authorized legal representative and a copy shall be maintained in the patient’s record file.

**DATA HANDLING AND RECORD KEEPING**

The CRFs must be completed in permanent black ink using a ballpoint pen. The form must be legible. Correction to data on a CRF may be made only by putting a single line through the incorrect data and writing the correct value, allowing the original text to remain legible. Each correction should be initialed and dated by the person making the change. If corrections are made after review and signature by the Investigator, he/she must be made aware of the changes and document this awareness. The consent forms and CRFs will be stored in a locked file cabinet in the PIs office.

**RECORDS RETENTION**

To comply with FDA regulations, the PI will maintain the records of drug disposition, patient exclusion logs, signed consent forms, CRFs, all correspondence, and supporting documentation for a minimum of 6 years after the investigation is complete. The Investigator may withdraw from the responsibility to maintain records and transfer custody of the records to another person who will accept responsibility for them. Notice of transfer must be given to the sponsor preferably before, but no later than, 10 days after transfer.

**DATA MONITORING**

During the course of the study, the sponsor or the IRB may conduct an on-site audit.

**CASE REPORT FORMS**

CRFs will be maintained in a locked file cabinet in the PIs office and kept current and up-to-date. Data should be entered within 24 hours of the patient’s evaluation. The CRF must be completed in black ballpoint pen. The investigator must sign and date all applicable pages.

**COMMUNICATION OF STUDY RESULTS**

The manuscript containing the overall study results will be distributed to the Sponsor for review before submission to a peer-reviewed journal, but the final contents will be at the discretion of the Principal Investigator(s). Any other manuscripts containing these data, including abstracts, must be distributed to the Sponsor before submission with a reasonable period for review. Submitted publications will conform to international standards for biomedical manuscripts, including authorship.

Reference List

(1) Wisplinghoff H, Bischoff T, Tallent SM, Seifert H, Wenzel RP, Edmond MB. Nosocomial bloodstream infections in US hospitals: analysis of 24,179 cases from a prospective nationwide surveillance study. Clin Infect Dis 2004; 39(3):309-317.

(2) Pfaller MA, Jones RN, Doern GV et al. International Surveillance of Bloodstream Infections Due to Candida Species: Frequency of Occurrence and Antifungal Susceptibilities of Isolates Collected in 1997áin the United States, Canada, and South America for the SENTRY Program. J Clin Microbiol 1998; 36(7):1886-1889.

(3) Falagas ME, Apostolou KE, Pappas VD. Attributable mortality of candidemia: a systematic review of matched cohort and case-control studies. Eur J Clin Microbiol Infect Dis 2006; 25(7):419-425.

(4) Fridkin SK. Candidemia is Costly--Plain and Simple. Clin Infect Dis 2005; 41(9):1240-1241.

(5) Zaoutis TE, Argon J, Chu J, Berlin JA, Walsh TJ, Feudtner C. The Epidemiology and Attributable Outcomes of Candidemia in Adults and Children Hospitalized in the United States: A Propensity Analysis. Clin Infect Dis 2005; 41(9):1232-1239.

(6) Cruciani M, de LF, Mengoli C. Prophylaxis of Candida infections in adult trauma and surgical intensive care patients: a systematic review and meta-analysis. Intensive Care Med 2005; 31(11):1479-1487.

(7) Lipsett PA. Clinical trials of antifungal prophylaxis among patients in surgical intensive care units: concepts and considerations. Clin Infect Dis 2004; 39 Suppl 4:S193-S199.

(8) Pelz RKM, Hendrix CWM, Swoboda SMR et al. Double-Blind Placebo-Controlled Trial of Fluconazole to Prevent Candidal Infections in Critically Ill Surgical Patients. [Article]. Annals of Surgery 2001; 233(4):542-548.

(9) Playford EG, Webster AC, Sorrell TC, Craig JC. Antifungal agents for preventing fungal infections in non-neutropenic critically ill and surgical patients: systematic review and meta-analysis of randomized clinical trials. J Antimicrob Chemother 2006; 57(4):628-638.

(10) Rex JH, Sobel JD. Prophylactic antifungal therapy in the intensive care unit. Clin Infect Dis 2001; 32(8):1191-1200.

(11) Rex JHM. Antifungal prophylaxis in the intensive care unit: Who should get it? *. [Editorial]. Critical Care Medicine 2006; 34(4):1286-1287.

(12) van Burik JA, Ratanatharathorn V, Stepan DE et al. Micafungin versus fluconazole for prophylaxis against invasive fungal infections during neutropenia in patients undergoing hematopoietic stem cell transplantation. Clin Infect Dis 2004; 39(10):1407-1416.

(13) Odabasi Z, Mattiuzzi G, Estey E et al. Beta-D-glucan as a diagnostic adjunct for invasive fungal infections: validation, cutoff development, and performance in patients with acute myelogenous leukemia and myelodysplastic syndrome. Clin Infect Dis 2004; 39(2):199-205.

(14) Mennink-Kersten MASH, Warris A, Verweij PE. 1,3-{beta}-D-Glucan in Patients Receiving Intravenous Amoxicillin-Clavulanic Acid. N Engl J Med 2006; 354(26):2834-2835.

(15) Kanda H, Kubo K, Hamasaki K et al. Influence of various hemodialysis membranes on the plasma (1[rarr]3)-[bgr]-D-glucan level. Kidney Int 2001; 60(1):319-323.

(16) Ogawa M, Hori H, Niiguchi S, Azuma E, Komada Y. False-positive plasma (1-->3)-beta-D-glucan test following immunoglobulin product replacement in an adult bone marrow recipient. Int J Hematol 2004; 80(1):97-98.

(17) Usami M, Ohata A, Horiuchi T, Nagasawa K, Wakabayashi T, Tanaka S. Positive (1?3)-beta-d-glucan in blood components and release of (1?3)-beta-d-glucan from depth-type membrane filters for blood processing. Transfusion 2002; 42(9):1189-1195.

(18) Marty FM, Lowry CM, Lempitski SJ, Kubiak DW, Finkelman MA, Baden LR. Reactivity of (1->3)-{beta}-D-Glucan Assay with Commonly Used Intravenous Antimicrobials. Antimicrob Agents Chemother 2006; 50(10):3450-3453.

(19) Sobel JD, Revankar SG. Echinocandins -- First-Choice or First-Line Therapy for Invasive Candidiasis? N Engl J Med 2007; 356(24):2525-2526.

(20) Vazquez JA, Sobel JD. Anidulafungin: A Novel Echinocandin. Clin Infect Dis 2006; 43(2):215-222.

(21) Dowell JA, Stogniew M, Krause D, Damle B. Anidulafungin Does Not Require Dosage Adjustment in Subjects With Varying Degrees of Hepatic or Renal Impairment. J Clin Pharmacol 2007; 47(4):461-470.

(22) Pfizer I. Eraxis Package Insert. 2007.
Ref Type: Generic

(23) Boldrick JC, Alizadeh AA, Diehn M et al. Stereotyped and specific gene expression programs in human innate immune responses to bacteria
1. Proc Natl Acad Sci U S A 2002; 99(2):972-977.

(24) Kawada J, Kimura H, Kamachi Y et al. Analysis of gene-expression profiles by oligonucleotide microarray in children with influenza
1. J Gen Virol 2006; 87(Pt 6):1677-1683.

(25) Ostrosky-Zeichner L, Alexander BD, Kett DH et al. Multicenter clinical evaluation of the (1-->3) beta-D-glucan assay as an aid to diagnosis of fungal infections in humans. Clin Infect Dis 2005; 41(5):654-659.

(26) Ascioglu S, Rex JH, de Pauw B et al. Defining Opportunistic Invasive Fungal Infections in Immunocompromised Patients with Cancer and Hematopoietic Stem Cell Transplants: An International Consensus
1. Clin Infect Dis 2002; 34(1):7.

(27) Takesue Y, Kakehashi M, Ohge H et al. Combined assessment of beta-D-glucan and degree of candida colonization before starting empiric therapy for candidiasis in surgical patients. World J Surg 2004; 28(6):625-630.

(28) Berchuck A, Iversen ES, Lancaster JM et al. Patterns of gene expression that characterize long-term survival in advanced stage serous ovarian cancers
1. Clin Cancer Res 2005; 11(10):3686-3696.

(29) Seo D, Wang T, Dressman H et al. Gene expression phenotypes of atherosclerosis
1. Arterioscler Thromb Vasc Biol 2004; 24(10):1922-1927.

(30) Wei C, Li J, Bumgarner RE. Sample size for detecting differentially expressed genes in microarray experiments
1. BMC Genomics 2004; 5(1):87.

**Table 1. Clinical Risk Factors for Invasive Candidiasis in Adult Intensive Care Patients**

| **Risk Factors** |
| --- |
| Prolonged length of stay |
| High acuity |
| Diabetes |
| Renal failure |
| Hemodialysis |
| Broad-spectrum antibiotics |
| Central venous catheter |
| Parenteral nutrition |
| Immunosuppressive drugs |
| Cancer chemotherapy |
| Severe acute pancreatitis |
| *Candida* colonization at multiple sites |
| Surgery |
| Transplantation |

**Table 2. Factors Associated with False Positive β-D-Glucan Test Results**

| Laboratory contamination  Non-glucan free glassware and/or plastics  Starched laboratory coats |
| --- |
| Immunoglobulin therapy |
| Albumin supplementation |
| Cellulose containing dialysis membranes |
| Serosal exposure to glucan containing gauze |
| Amoxicillin-clavulonic acid therapy |

**Table 3. Definitions of Invasive Fungal Infections**

| **Category, type of infection** | **Description** |
| --- | --- |
| Proven IFIs |  |
| Deep tissue infections |  |
| Molds | Histopathologic or cytopathologic examination showing hyphae from needle aspiration or biopsy specimen with evidence of associated tissue damage (either microscopically or unequivocally by imaging); or positive culture result for a sample obtained by sterile procedure from normally sterile and clinically or radiologically abnormal site consistent with infection, excluding urine and mucous membranes. |
| Yeasts | Histopathologic or cytopathologic examination showing yeast cells (*Candida* species may also show pseudohyphae or true hyphae) from specimens of needle aspiration or biopsy excluding mucous membranes; or positive culture result on sample obtained by sterile procedure from normally sterile and clinically or radiologically abnormal site consistent with infection, excluding urine, sinuses, and mucous membranes; or microscopy (India ink, mucicarmine stain) or antigen positivity for *Cryptococcus* species in CSF. |
| Fungemia |  |
| Molds | Blood culture that yields fungi, excluding *Aspergillus* species and *Penicillium* species other than *Penicillium marneffei*, accompanied by temporally related clinical signs and symptoms compatible with relevant organism. |
| Yeasts | Blood culture that yields *Candida* species and other yeasts in patients with temporally related clinical signs and symptoms compatible with relevant organism. |
| Systemic or confined to lungs | Must be proven by culture from site affected, in host with symptoms attributed to fungal infection; if culture results are negative or unattainable, histopathologic or direct microscopic demonstration of appropriate morphological forms is considered adequate for dimorphic fungi (*Blastomyces, Coccidioides* and *Paracoccidioides* species) having truly distinctive appearance; *Histoplasma capsulatum* variant *capsulatum* may resemble *Candida* *glabrata*. |
| Disseminated | May be established by positive blood culture result or positive result for urine or serum antigen by means of RIA [17] |
| Probable IFIs | At least 1 host factor criterion (see Table4); and 1 microbiological criterion; and 1 major (or 2 minor) clinical criteria from abnormal site consistent with infection. |
| Possible IFIs^d^ | At least 1 host factor criterion; and 1 microbiological or 1 major (or 2 minor) clinical criteria from abnormal site consistent with infection. |

**(Adapted from Ascioglu et al. (26)**

**Table 4. Host Factors, Microbiological, and Clinical Criteria for Invasive Fungal Infections**

| **Type of criteria** | **Criteria** |
| --- | --- |
| Host factors | Neutropenia (<500 neutrophils/mm^3^ for >10 days) |
|  | Persistent high fever for >96 h refractory to appropriate broad-spectrum antibacterial treatment in high-risk patients |
|  | Body temperature either >38°C and any of the following predisposing conditions: prolonged neutropenia (>10 days) in previous 60 days, recent or current use of significant immuno-suppressive agents in previous 30 days, proven or probable IFI during previous episode or neutropenia, or coexistence of symptomatic AIDS |
|  | Signs and symptoms indicating graft-versus-host disease, particularly severe (grade ≥2) or chronic extensive disease |
|  | Prolonged (>3 weeks) use of corticosteroids in previous 60 days |
| Microbiological | Positive result of culture for mold (including *Aspergillus, Fusarium,* or *Scedosporium* species or Zygomycetes) or *Cryptococcus neoformans* or an endemic fungal pathogen^a^ from sputum or bronchoalveolar lavage fluid samples |
|  | Positive result of culture or findings of cytologic/direct microscopic evaluation for mold from sinus aspirate specimen |
|  | Positive findings of cytologic/direct microscopic evaluation for mold or *Cryptococcus* species from sputum or bronchoalveolar lavage fluid samples |
|  | Positive result for *Aspergillus* antigen in specimens of bronchoalveolar lavage fluid, CSF, or ≥2 blood samples |
|  | Positive result for cryptococcal antigen in blood sample |
|  | Positive findings of cytologic or direct microscopic examination for fungal elements in sterile body fluid samples (e.g., *Cryptococcus* species in CSF) |
|  | Positive result for *Histoplasma capsulatum* antigen in blood, urine, or CSF |
|  | Two positive results of culture of urine samples for yeasts in absence of urinary catheter |
|  | *Candida* casts in urine in absence of urinary catheter |
|  | Positive result of blood culture for *Candida* species |
| Clinical | Must be related to site of microbiological criteria and temporally related to current episode |
| Lower respiratory tract infection |  |
| Major | Any of the following new infiltrates on CT imaging: halo sign, air-crescent sign, or cavity within area of consolidation^c^ |
| Minor | Symptoms of lower respiratory tract infection (cough, chest pain, hemoptysis, dyspnea); physical finding of pleural rub; any new infiltrate not fulfilling major criterion; pleural effusion |
| Sinonasal infection |  |
| Major | Suggestive radiological evidence of invasive infection in sinuses (i.e., erosion of sinus wall or extension of infection to neighboring structures, extensive skull base destruction) |
| Minor | Upper respiratory symptoms (e.g., nasal discharge, stuffiness); nose ulceration or eschar of nasal mucosa or epistaxis; periorbital swelling; maxillary tenderness; black necrotic lesions or perforation of hard palate |
| CNS infection |  |
| Major | Radiological evidence suggesting CNS infection (e.g., mastoiditis or other parameningeal foci, extradural empyema, intraparenchymal brain or spinal cord mass lesion) |
| Minor | Focal neurological symptoms and signs (including focal seizures, hemiparesis, and cranial nerve palsies); mental changes; meningeal irritation findings; abnormalities in CSF biochemistry and cell count (provided that CSF is negative for other pathogens by culture or microscopy and negative for malignant cells) |
| Disseminated fungal infection | Papular or nodular skin lesions without any other explanation; intraocular findings suggestive of hematogenous fungal chorioretinitis or endophthalmitis |
| Chronic disseminated candidiasis | Small, peripheral, targetlike abscesses (bull’s-eye lesions) in liver and/or spleen demonstrated by CT, MRI, or ultrasound, as well as elevated serum alkaline phosphatase level; supporting microbiological criteria are not required for probable category |
| Candidemia | Clinical criteria are not require for probable candidemia; there is no definition for possible candidemia |

^a^*H. capsulatum* variant *capsulatum*, *Blastomyces dermatitidis*, *Coccidioides immitis*, or *Paracoccidioides brasiliensis*.. ^b^In absence of infection by organisms that may lead to similar radiological findings including cavitation, such as *Mycobacterium*, *Legionella*, and *Nocardia* species. **Adapted from Ascioglu et al.(26)**

**Table 5. Clinical Response**

| **Clinical response*** | **Definition** |
| --- | --- |
| Response to empiric therapy | Resolution of fever and/or other attributable symptoms/signs of IFI |
| Response to preemptive therapy | Resolution of abnormal BDG test result |

*Clinical response as an efficacy endpoint will be assessed at the end of treatment.

**Figure 1. Study Design**
